# Clinical, Radiological, and Functional Outcomes of Tableless Direct Anterior Total Hip Arthroplasty: A Single-Center Experience From Hospital Sultanah Bahiyah, Malaysia

**DOI:** 10.7759/cureus.106281

**Published:** 2026-04-01

**Authors:** Mohd Aizat Azfar Soldin, Muhammad Azhar Abdullah, Ahmad Fauzey Kassim, Mawardi B Mustapha, Muhindra Rao Gsangaya, Muhammad Zainal Abidin

**Affiliations:** 1 Orthopedics and Traumatology, Ministry of Health of Malaysia, Selangor, MYS; 2 Orthopedics, Hospital Sultanah Bahiyah, Alor Setar, MYS; 3 Orthopedics, Hospital Raja Perempuan Zainab II, Kota Bharu, MYS; 4 Community and Family Medicine, Kulliyyah of Medicine, Kuantan, MYS

**Keywords:** direct anterior approach, functional outcome, minimally invasive, tableless technique, total hip arthroplasty

## Abstract

Introduction

The direct anterior approach (DAA) is underutilized in Malaysia due to limited surgeon exposure and the perceived need for specialized traction tables despite its documented advantages. This study evaluates short-term clinical, radiological, and functional outcomes of tableless DAA total hip arthroplasty (THA) performed using a standard operating table in a Malaysian tertiary center.

Methodology

This retrospective cohort case series included all nine patients (10 hips) who underwent primary DAA THA from October 2023 to October 2024. Outcomes assessed were pain scores, mobilization time, complications, acetabular orientation, femoral offset, limb-length discrepancy (LLD), subsidence, and functional status via Harris Hip Score (HHS) during follow-up at one, three, and six months. Descriptive statistics and non-parametric tests (Friedman, Cochran Q) were applied.

Results

Median age was 58 years, and median body mass index (BMI) was 24.8 kg/m². Pain improved rapidly (visual analog scale (VAS) score: 1.4 on day 1 to 0.8 on day 3). Most patients mobilized with support by day 2, and seven patients were discharged by day 3. Complications included two intraoperative greater trochanteric fractures (both tension-band wired) and one superficial infection. No dislocations, lateral femoral cutaneous nerve (LFCN) palsy, deep vein thrombosis/venous thromboembolism (DVT/VTE), subsidence, or implant loosening were observed. Radiographs demonstrated median cup inclination of 44.1° (44.0°-45.2°), median femoral offset difference of 2.0 mm, and mean LLD of 3.0 mm. Median HHS improved from 42 preoperatively to 92 at six months (*P* < 0.001).

Conclusions

Tableless DAA THA performed on a standard operating table is safe, reproducible, and yields favorable early outcomes comparable to international DAA benchmarks. These findings support its wider adoption in resource-limited Malaysian arthroplasty centers.

## Introduction

Total hip arthroplasty (THA) is widely regarded as one of the most effective surgical procedures for end-stage hip disorders, providing pain relief, restoration of function, and improved quality of life [1-4]. Although the overall success of THA is well established, the choice of surgical approach remains an area of ongoing debate. The posterolateral approach (PA) has historically been the most widely used, while the lateral approach remains commonly practiced in several regions, including Malaysia [5].

The direct anterior approach (DAA) has gained increasing attention due to its muscle-sparing technique, utilizing an internervous and intermuscular plane. Compared with conventional approaches, DAA has been associated with reduced soft tissue trauma, faster early functional recovery, lower dislocation rates, and shorter hospital stay [6-9]. Meta-analyses of randomized controlled trials (RCTs) have demonstrated improved short-term outcomes, including pain and functional scores, although these benefits are accompanied by longer operative times and a steep learning curve [1,3,7]. However, DAA is not without limitations. It carries a distinct complication profile, including lateral femoral cutaneous nerve (LFCN) neuropraxia, intraoperative femoral fractures, wound-related complications, and technical challenges related to femoral exposure, particularly during the early learning phase. These factors contribute to its technical complexity and may limit widespread adoption, especially in low-volume centers [2].

In Asia, emerging evidence supports the feasibility and benefits of DAA. A propensity-matched study from Singapore demonstrated shorter hospital stays, improved functional outcomes, and lower dislocation rates compared with PA [8,10]. Despite these encouraging findings, the adoption of DAA in Malaysia remains limited. This is likely multifactorial, including limited exposure during local training, a shortage of surgeons with formal experience in DAA, and the perceived dependence on specialized traction tables such as the HANA® table, which may not be readily available due to cost constraints. Additionally, the lack of locally published outcome data has further limited confidence and uptake among surgeons [5].

Recent literature has demonstrated that DAA can be safely and effectively performed using a standard operating table, which is particularly relevant in resource-limited settings [11]. Specialized traction tables are designed to facilitate controlled limb positioning, including extension, adduction, and external rotation, thereby improving femoral exposure during preparation. They also allow stabilization and independent manipulation of the operative limb, aiding elevation of the proximal femur and reducing the need for manual assistance. However, these systems are costly and not universally available. The *tableless* adaptation may, therefore, represent a practical solution to overcome this barrier and support wider adoption of DAA in Malaysia.

Against this background, this retrospective cohort study aims to assess the clinical, radiological, and functional outcomes of DAA on conventional operating table THA performed at Hospital Sultanah Bahiyah, Kedah, providing valuable data to guide the wider acceptance and practice of this approach in Malaysia.

## Materials and methods

Methodology

This retrospective cohort case series included all nine patients (10 hips) who underwent primary total hip arthroplasty using the DAA at Hospital Sultanah Bahiyah, Alor Setar. This case series was registered and approved under the National Medical Research Register (NMRR-25-03649-BYB). As this study represents our early experience with the DAA, patient selection was deliberately restricted to minimize complications during the learning phase. Inclusion criteria were patients aged 18 to 80 years with end-stage osteoarthritis or other hip pathologies requiring total hip arthroplasty, deemed suitable for DAA based on preoperative assessment, with a body mass index (BMI) <30 kg/m², good bone quality (absence of osteoporosis or osteopenia), and willingness to provide informed consent and comply with follow-up. All surgeries were performed by two certified arthroplasty surgeons using the DAA on a standard operating table.

**Figure 1:**
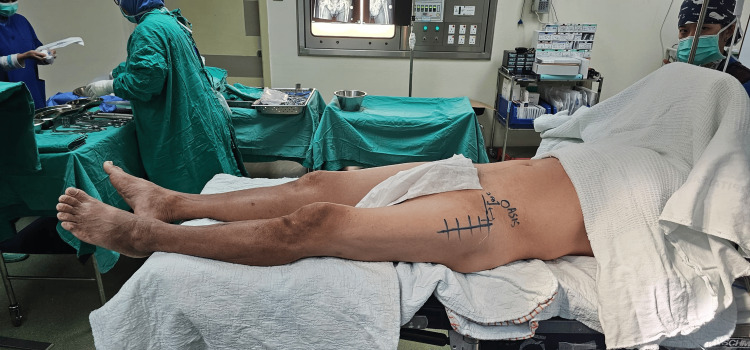
Supine position on a conventional operating table.

**Figure 2:**
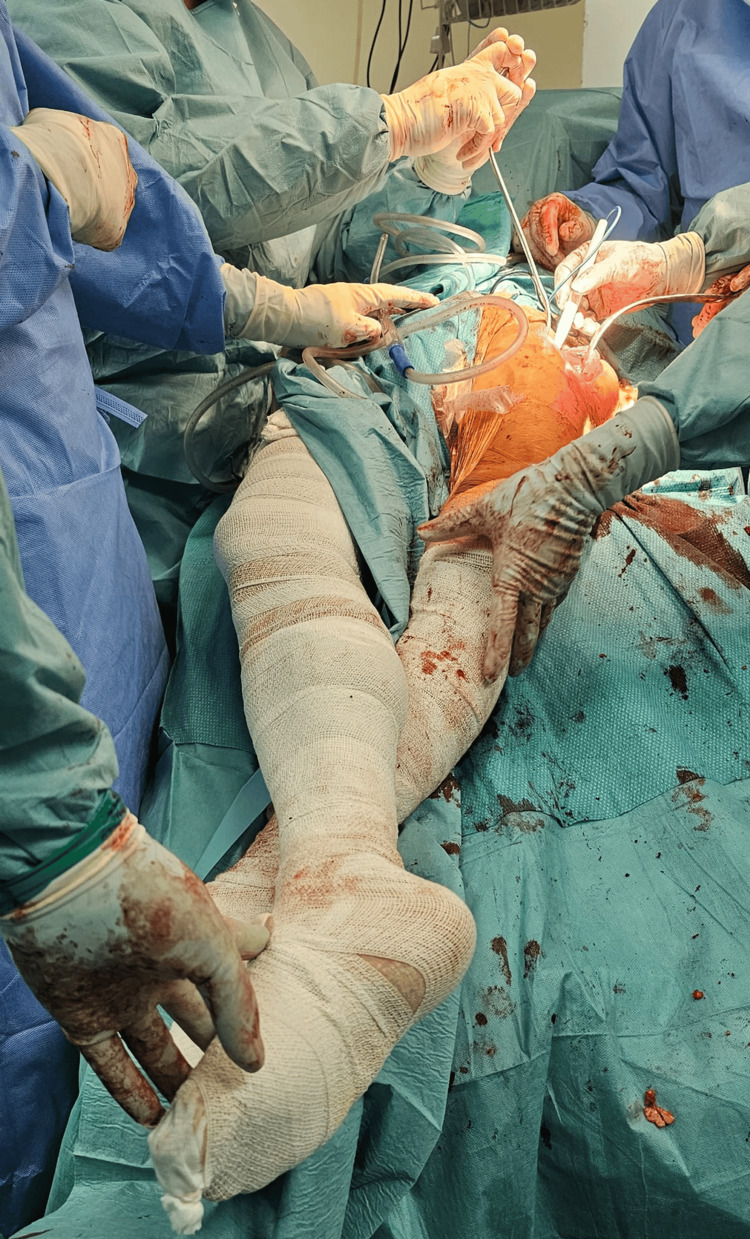
Position of the operated limb during femoral preparation. The operative limb was positioned in extension, external rotation, and maximal adduction by crossing it below the contralateral limb during femoral broaching (extension and external rotation are not depicted in this image).

Patients were positioned supine with the operative hip aligned to the pivot point of the table to allow adduction and external rotation of the limb for femoral exposure. Both lower limbs were prepped and draped. Anatomical landmarks, including the anterior superior iliac spine (ASIS), greater trochanter, and patella, were identified and marked (Figure 1). An initial 15-cm incision was made, beginning approximately two finger breadths distal and lateral to the ASIS and directed toward the lateral aspect of the patella. The fascia overlying the tensor fascia lata (TFL) was identified and incised in line with the muscle fibers. The intermuscular plane between the sartorius and TFL was developed. The ascending branch of the lateral femoral circumflex artery was identified and ligated. The rectus femoris and iliocapsularis were retracted medially to expose the capsule. A T-shaped capsulotomy was performed, followed by placement of stay sutures. The femoral neck was osteotomized as templated, and the head was removed. The acetabulum was sequentially reamed under fluoroscopic guidance, and the cup was implanted, ensuring appropriate inclination and anteversion. A trial liner was placed. With adduction, external rotation, and extension of the lower limb, inferior (pubofemoral) and superior capsular releases were performed. During femoral preparation, the operative limb was positioned in extension, external rotation, and maximal adduction by crossing it below the contralateral limb. This maneuver is performed intraoperatively to facilitate delivery of the proximal femur and improve exposure for broaching (Figure 2). Adequate capsular release, particularly of the superior capsule, allows controlled mobilization of the femur without excessive force. A curved bone spike was used beneath the greater trochanter to aid femoral elevation. Offset broaches were used for femoral canal preparation, followed by stem implantation and stability assessment. The capsule, fascia, subcutaneous tissue, and skin were closed in layers. 

All procedures were conducted under combined spinal epidural (CSE) anesthesia. Before wound closure, periarticular infiltration was administered using the Ranawat multimodal injection cocktail, comprising adrenaline (0.3 mL of 1:1,000), bupivacaine 0.5% (24 mL), cefuroxime 750 mg, morphine 8 mg (0.8 mL), and normal saline (methylprednisolone and ketorolac were excluded). This multimodal analgesic protocol is widely utilized in arthroplasty to optimize postoperative pain control, reduce opioid consumption, and support early rehabilitation. The CSE catheter was maintained until the morning of the first postoperative day. Postoperative analgesia consisted of intravenous parecoxib 40 mg twice daily on postoperative day 1, followed by oral celecoxib 200 mg twice daily and paracetamol 1 g four times daily for the subsequent days until discharge.

Pain was evaluated using the visual analog scale (VAS). Time to mobilization, total operative duration, and intraoperative complications (e.g., periprosthetic fractures) were recorded. Postoperative complications, including LFCN palsy, deep vein thrombosis (DVT), infection, dislocation, and mortality, were also documented.

Standard anteroposterior pelvic radiographs were evaluated to determine acetabular cup inclination and anteversion, femoral offset, limb length, and any evidence of implant subsidence. Functional outcomes were assessed using the Harris Hip Score (HHS) [12] preoperatively and at one, three, and six months.

Descriptive statistics were used to summarize demographic and outcome data. Continuous variables were reported as median with interquartile range (IQR). Categorical variables were expressed as frequencies and percentages. Comparative analysis was performed against published benchmarks of DAA THA performed using specialized traction tables, focusing on clinical, radiological, and functional outcomes. The Friedman test was conducted to compare the median of HHS between follow-up periods. The Cochran Q test was conducted to compare mobility outcomes between follow-up periods. A *P*-value <0.001 was considered statistically significant. 

## Results

Nine patients (10 hips) underwent THA using the tableless DAA. The cohort comprised three males (33.3%) and six females (66.7%), with a median age of 58 years (range = 32-74 years). The median BMI was 24.8 kg/m² (range = 17.2-28.3 kg/m²). Surgical indications included primary osteoarthritis (*n* = 2), rheumatoid arthritis (*n* = 2), seronegative rheumatoid arthritis (*n* = 1), bilateral avascular necrosis (*n* = 1), avascular necrosis secondary to previous screw fixation (*n* = 1), neglected neck of femur fracture (*n* = 1), and acute femoral neck fracture (*n *= 1). Fixation methods consisted of one hybrid fixation (11.1%) and eight uncemented implants (88.9%) (Table 1).

**Table 1:** Characteristics of patients (N = 9). BMI, body mass index; AVN, avascular necrosis

Patient	Age (years)	Gender	BMI	Diagnosis	Fixations
1	61	Male	27.0 kg/m^2^	Neck of femur fracture (NOF)	Uncemented
2	32	Female	25.3 kg/m^2^	Osteoarthritis (OA)	Hybrid
3	59	Male	22.0 kg/m^2^	Rheumatoid arthritis (RA)	Uncemented
4	74	Female	24.8 kg/m^2^	Seronegative RA	Uncemented
5	64	Female	28.3 kg/m^2^	Bilateral AVN	Uncemented
6	58	Female	21.5 kg/m^2^	Osteoarthritis	Uncemented
7	56	Female	23.4 kg/m^2^	Neglected NOF	Uncemented
8	57	Male	26.0 kg/m^2^	AVN secondary to screw fixation	Uncemented
9	37	Female	17.2 kg/m^2^	OA secondary to screw fixation	Uncemented

**Figure 3:**
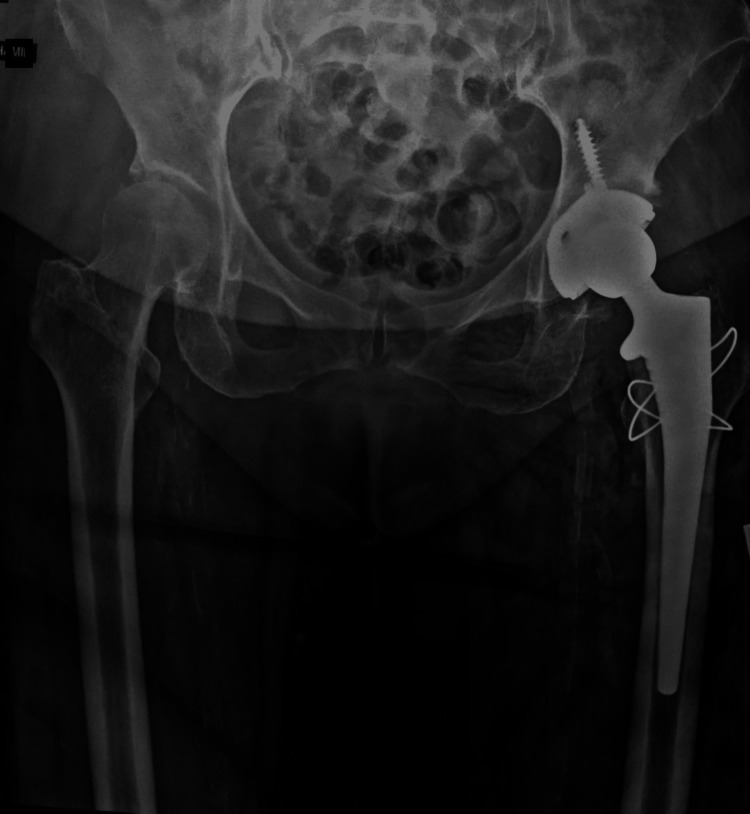
Postoperative X-ray of patient 3 with iatrogenic greater tuberosity fracture and managed with tension band wiring.

**Figure 4:**
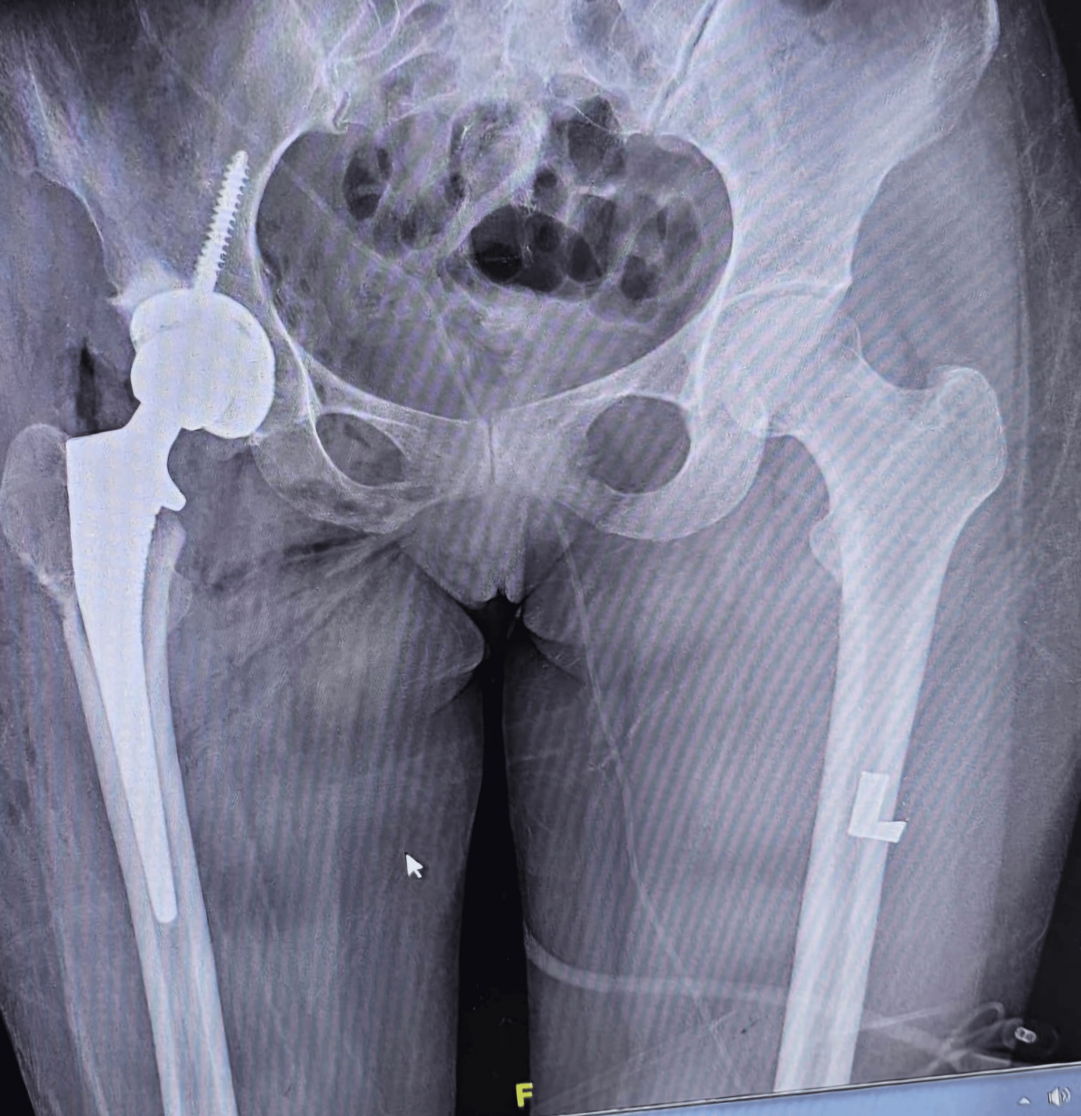
Postoperative X-ray of patient 9.

Postoperative pain improved rapidly, with the mean VAS score declining from 1.4 on day 1 to 0.8 on day 3. Most patients mobilized with support by postoperative day 2; however, three patients (Cases 4, 8, and 9) began mobilization on day 1. The majority of patients were discharged on postoperative day 3, except Cases 2 and 8, whose discharges were delayed due to uncontrolled diabetes mellitus. One patient developed a superficial surgical site infection at 10 days postoperatively, which resolved completely after three days of intravenous antibiotics. No other complications, including LFCN palsy, dislocation, limb length discrepancy, or foot drop, were observed. Apart from that, no obvious or documented complications attributable to prolonged anesthesia or operative time, such as thromboembolic events, wound complications, or anesthesia-related adverse events, were observed (Table 2, Figures 3-4). 

**Table 2:** Surgical and radiological outcomes (N = 9). *Median (Range). DVT/PE, deep vein thrombosis/pulmonary embolism

Outcome		*n* (%)
Surgical	Iatrogenic fractures	2 (22.2%)
	Surgery time (mean)	188 minutes
	VAS	
	Day 1 (mean)	1.4
	Score 1	5 (50.5%)
	Score 2	4 (44.4%)
	Day 2 (mean)	1
	Score 1	9 (100%)
	Score 2	0 (0%)
	Day 3 (mean)	0.8
	Score 0	2 (22.2%)
	Score 1	7 (77.7%)
	Mobilization with support	
	Day 1	3 (33.3%)
	Day 2	6 (66.6%)
	Discharge	
	Day 3	7 (77.7%)
	Day 5	1 (11.1%)
	Day 7	1 (11.1%)
	Postoperative infection	1 (11.1%)
	Lateral cutaneous femoral nerve injury and DVT/PE, foot drop	0 (0%)
Radiological	Acetabular inclination	44.1^o^ (44.0,45.2)*
	Femoral offset difference (mm)	2.0 mm (1,5)*
	Limb length discrepancy (mm)	3 (1,20)*
	Subsidence	0
	Loosening	0

**Figure 5:**
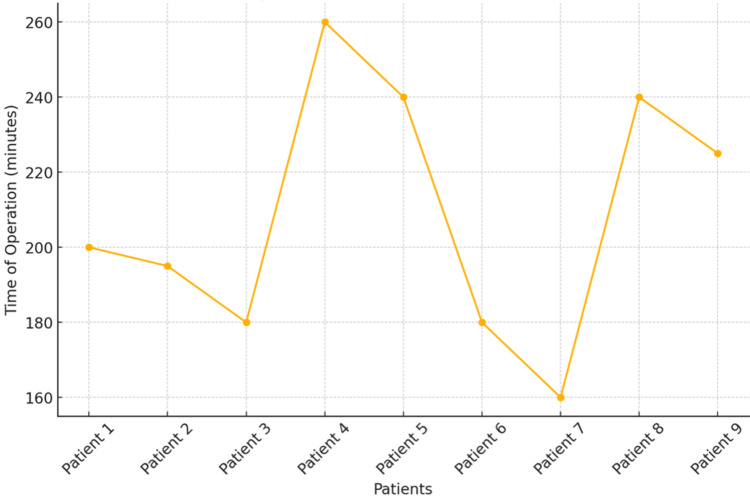
Operating time.

The mean operative time was 188 minutes (SD = 33.8, range = 160-260 minutes). Surgical time decreased progressively with subsequent cases, reflecting improvement along the learning curve. Prolonged operative times were observed in patients requiring bilateral procedures, additional fixation (greater trochanteric fractures), or removal of prior implants. Intraoperatively, two patients sustained greater trochanteric fractures, both treated successfully with tension band wiring (Figure 5).

Radiographic evaluation demonstrated a median acetabular inclination of 44.1°, within the recognized safe zone. The median femoral offset difference was 2.0 mm, and the median limb length discrepancy was 3.0 mm. No cases of stem subsidence or implant loosening were observed at six months’ follow-up.

**Figure 6:**
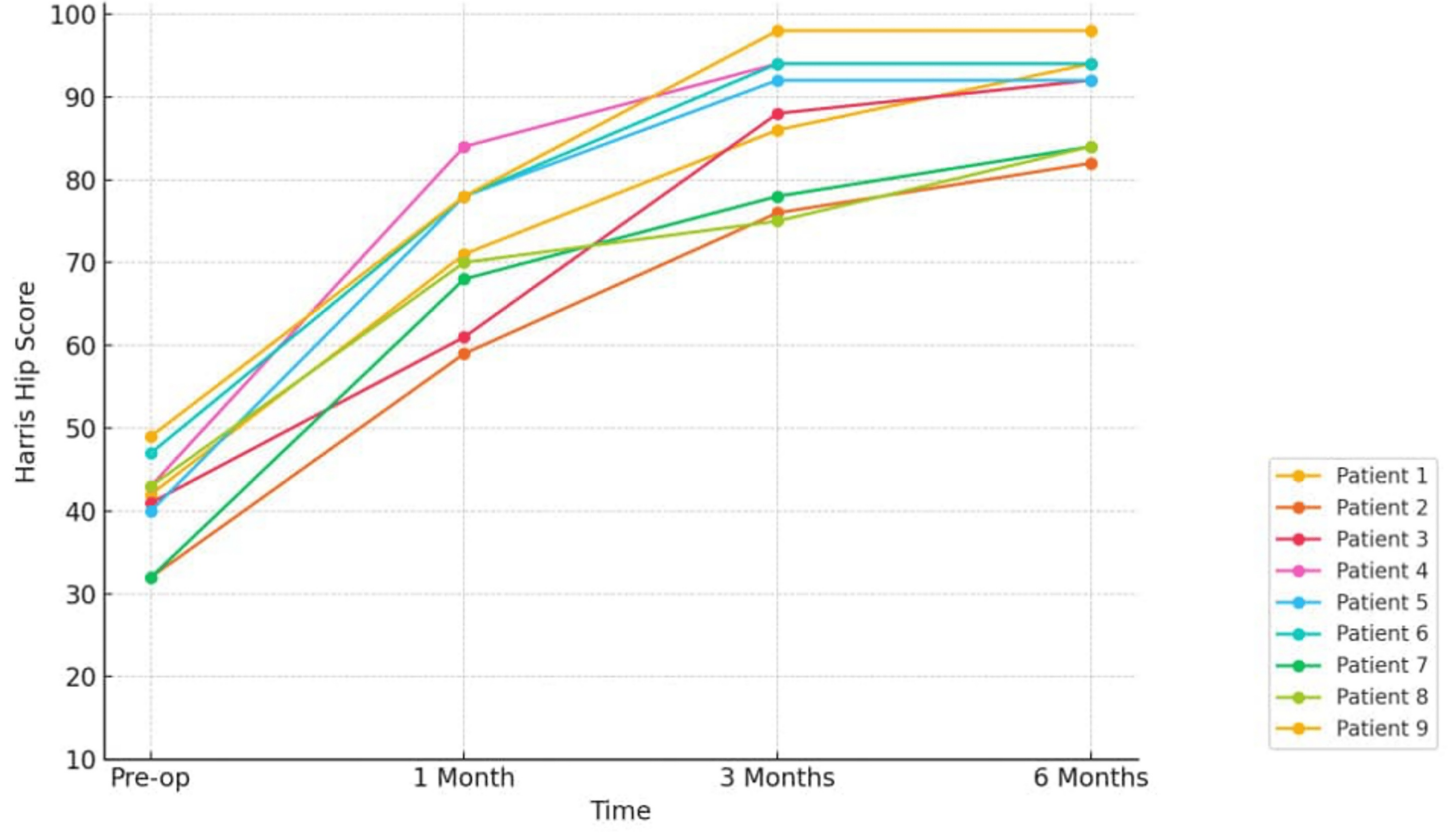
Harris Hip Score progression over time.

Functional recovery demonstrated progressive improvement over time (Figure 6). Median HHS increased from 42 (IQR 32-49) preoperatively to 71 (IQR 61-84) at one month, 88 (IQR 75-98) at three months, and 92 (IQR 84-98) at six months. Repeated-measures analysis using the Friedman test revealed a statistically significant difference across time points (χ² = 25.133, *P* < 0.001), indicating consistent functional improvement. Clinically, four patients achieved excellent functional scores by three months, and by six months, six patients (67%) demonstrated excellent outcomes, with the remaining patients achieving good scores (Table 3).

**Table 3:** Functional outcomes (N = 9). *P-value significant at <0.001. †Friedman test. ‡Cochran Q test (aided vs. unaided).

Outcome	Pre-op	1 month	3 months	6 months	Test statistic	*P*-value
HHS, median (IQR)	42 (32-49)	71 (61-84)	88 (75-98)	92 (84-98)	25.133^†^	<0.001*
Mobility, *n* (%)					15.0^‡^	<0.001*
Wheelchair	3 (33.3%)	0	0	0		
Crutches	1 (11.1%)	0	0	0		
Walking frame	1 (11.1%)	6 (66.6%)	2 (22.2%)	0		
Cane	4 (44.4%)	2 (22.2%)	3 (33.3%)	2 (22.2%)		
Unaided	0	1 (11.1%)	4 (44.4%)	7 (77.7%)		

Mobility status improved progressively throughout follow-up. Mobility status was recategorized into aided (wheelchair, crutches, walking frame, and cane) and unaided, and repeated-measures analysis using the Cochran Q test showed statistical significance. While several patients required assistive devices during the early postoperative period, seven patients (77.7%) were ambulating unaided at six months. However, this must be interpreted with caution due to the small sample size and sparse distribution.

## Discussion

Between October 2023 and October 2024, a total of 57 primary THAs were performed at Hospital Sultanah Bahiyah, comprising 42 procedures via the lateral approach, 5 via the posterior approach, and 10 via the DAA. As this study represents our early experience with the DAA, patient selection was deliberately restricted to minimize complications during the learning phase. Inclusion criteria were patients aged 18 to 80 years with end-stage osteoarthritis or other hip pathologies requiring THA, deemed suitable for DAA based on preoperative assessment, with a BMI < 30 kg/m², good bone quality (absence of osteoporosis or osteopenia), and willingness to provide informed consent and comply with follow-up. As surgical experience increases and familiarity with the technique improves, the indications and patient selection criteria may be progressively expanded to include more complex cases, including patients with higher BMI, more challenging anatomy, and reduced bone quality.

Despite its technical complexity and steep learning curve, the DAA has been associated with faster functional recovery, shorter hospital stay, and lower dislocation rates when compared to conventional approaches [1,7]. The DAA is considered technically demanding due to its limited surgical exposure through a muscle-sparing interval, where no muscles are detached. This necessitates precise identification of anatomical planes, careful soft tissue handling, and selective capsular releases to achieve adequate exposure. In addition, femoral preparation can be particularly challenging, requiring specific limb positioning maneuvers, specialized instruments such as offset broaches, and a thorough understanding of the surgical technique. These factors contribute to the steep learning curve and increased technical complexity of the approach.

In our series, operative time was initially prolonged for DAA cases, especially in patients requiring bilateral procedures, revision implant removal, or fixation of concomitant greater trochanteric fractures. Prolonged operative and anesthetic times during the initial learning phase of the DAA may potentially increase the risk of perioperative complications. This underscores the importance of appropriate patient selection and gradual skill acquisition during the adoption of this technique. However, operative duration improved progressively with case experience, reflecting the learning curve effect. Similar findings have been consistently reported in the literature, with early series documenting an additional 15-20 minutes of operating time for DAA relative to posterior approaches [1,7]. Importantly, these longer operative times tend to normalize after approximately 50-100 cases, indicating that proficiency is attainable with structured training and careful patient selection [10]. 

Intraoperatively, we encountered two cases of greater trochanteric fracture, both successfully managed with tension band wiring, an observation aligned with other reports noting an increased risk of intraoperative fracture during the initial learning phase of DAA [10]. Despite these technical challenges, the absence of LFCN injury in our series is notable, as prior studies have documented incidences ranging from 5% to 11% [13]. We attribute this to meticulous dissection and avoidance of an excessively lateral plane beyond the line from the anterior superior iliac spine to the lateral patella.

In our tableless DAA series, perioperative complications were low, two intraoperative greater trochanteric fractures (both tension-band wired with uneventful recovery) and one superficial surgical site infection that resolved with a short course of intravenous antibiotics. Postoperatively, no dislocations, no LFCN palsy, and no clinically relevant limb length problems were observed at early follow-up. These findings align with contemporary evidence. Insufficient exposure of the proximal femur has been associated with an increased risk of iatrogenic fractures. The combination of appropriate soft tissue release, controlled limb positioning, and the use of offset broaches facilitates safe femoral preparation while minimizing complications. In our series, no obvious or documented complications attributable to prolonged anesthesia or operative time, such as thromboembolic events, wound complications, or anesthesia-related adverse events, were observed. However, this should be interpreted with caution, given the small sample size. A level-I meta-analysis limited to RCTs reported no significant differences in dislocation, periprosthetic fracture, or venous thromboembolism (VTE) when comparing DAA with posterolateral or lateral approaches, while confirming longer operative times and better early function with DAA [1]. Likewise, an updated RCT-only synthesis found better early pain/function after DAA with a trade-off of longer operative time, but no signal for increased thromboembolic events [7]. In an Asian propensity-matched cohort, DAA had a shorter length of stay and no dislocations, whereas a single (1.9%) posterior-approach dislocation required revision, consistent with the intrinsically stable, muscle-sparing nature of DAA [8].

Median limb-length discrepancy (LLD) in our cohort was 3 mm, well within clinically acceptable thresholds. The supine positioning inherent to DAA, coupled with routine intraoperative fluoroscopy, facilitates more accurate leg length restoration by enabling direct pelvic levelling and reproducible bony landmarks throughout broaching and trialing. This mechanistic advantage is congruent with reports of more accurate component positioning and early functional gains with DAA in regional data [8,14]. Our results strengthen this evidence, confirming that precise limb length restoration can be achieved without traction tables.

Dislocation remains a principal concern in THA. Evidence from both Asian and Western cohorts confirms substantially lower dislocation rates with DAA compared to posterior approaches [2,8]. Consistent with this, no dislocations occurred in our DAA cohort. Our findings also mirror the 2025 JBJS multicenter study, which reported a cumulative incidence of only 0.14% revision for instability at five years [1]. These consistent results underscore the intrinsic stability advantage of DAA and likely contribute to earlier patient confidence and ambulation without walking aids, as supported by multiple RCTs and meta-analyses [1,7,9].

Radiographically, all cups were within the accepted safe-zone inclination, with a median of 44.1°, and femoral offset was restored (median difference 2.0 mm). No subsidence or loosening was observed at six months. These radiographic results likely reflect the synergy of supine positioning and intraoperative imaging, which together aid consistent acetabular orientation and offset/length calibration. This is consistent with contemporary series showing accurate component positioning and early functional recovery after DAA, even in Asian centers transitioning to the technique [8,14,15].

In our DAA cohort, early HHS improvements were evident at six weeks, consistent with meta-analyses showing superior early function with DAA compared to both posterior and lateral approaches (mean difference, MD = +8 at six weeks vs. posterior; MD = +2 at 12 weeks vs. lateral) [1,7]. These findings are consistent with a Singaporean case series, which reported improved patient-reported outcomes and a shorter length of hospital stay in patients undergoing THA via the DAA compared with the posterior approach [8]. Our results, therefore, appear comparable, reinforcing that DAA can provide early clinical benefit even without specialized traction tables.

While our follow-up remains short, international data are reassuring. The UK National Joint Registry analysis (723,904 cases) found that anterior approaches performed comparably to posterior in terms of long-term implant survival, whereas lateral approaches were associated with slightly higher revision and mortality rates [2]. Moreover, long-term data have reported approximately 96% survivorship for DAA THA at 10 years, with instability-related revision being exceedingly rare [1]. However, obesity (BMI ≥40) remains a risk factor for adverse outcomes, including periprosthetic joint infection and reoperation, emphasizing the importance of patient selection in DAA [1].

Overall, our complication profile (low infection, no VTE, no dislocation, minimal LLD), radiographic accuracy, and functional recovery are concordant with RCT-level and regional data supporting DAA’s early functional advantages without increased major complication risk. Operative times were initially longer, consistent with global experience, but improved along the learning curve. Our absence of dislocations and low LLD corroborates the key theoretical advantages of DAA, while the use of standard tables with intraoperative imaging demonstrates that these outcomes can be achieved without expensive equipment, an important consideration in cost-constrained healthcare systems such as Malaysia. 

This study has several limitations. The sample size was small, reflecting our early institutional experience with the tableless DAA, which may limit statistical power. In addition, follow-up duration was limited to six months, restricting evaluation of long-term functional outcomes, implant survivorship, and late complications. Patient selection was intentionally restricted during the initial adoption phase to minimize complications and ensure procedural safety. Inclusion criteria focused on selected primary THA cases with favorable anatomy and pathology, consistent with a cautious implementation strategy during the learning curve. As surgical experience increases and familiarity with the technique improves, these criteria may be progressively expanded to include more complex cases. Future studies with larger cohorts, extended follow-up, and broader inclusion criteria will be important to validate outcomes and assess the wider applicability of the tableless DAA in Malaysian practice.

## Conclusions

This case series suggests that the DAA for THA can be safely performed in a Malaysian tertiary center using a standard operating table without specialized traction equipment. In this early experience, the tableless technique demonstrated favorable short-term clinical, radiological, and functional outcomes, with low complication rates, no observed dislocations or thromboembolic events, and acceptable restoration of limb length and component positioning.

However, these findings should be interpreted with caution, given the small sample size, short duration of follow-up, and selective patient cohort during the initial learning phase. As such, the results represent early outcomes rather than definitive conclusions.

Nevertheless, this study highlights the feasibility of tableless DAA in a resource-limited setting and may support its adoption with appropriate training and patient selection. Further studies with larger cohorts, longer follow-up, and broader patient inclusion are required to validate these findings and establish the generalizability of this technique in routine clinical practice.
